# Bilateral Neurofibromas of the Nipple-Areolar Complex: A Case Report and Approach to Diagnosis

**DOI:** 10.1155/2018/6702561

**Published:** 2018-09-06

**Authors:** Emily Hero, Martyn Carey, Isabelle Hero, Abeer M. Shaaban

**Affiliations:** ^1^University Hospitals Coventry and Warwickshire NHS Trust, UK; ^2^Department of Cellular Pathology, Queen Elizabeth Hospital Birmingham, UK

## Abstract

Neurofibromatosis type 1 is an autosomal dominant condition which can manifest as multiple neurofibromas within subcutaneous tissue. Neurofibromas of the breast are rare and most often encountered on the nipple-areolar complexes. A 33-year-old woman presented with large, bilateral, fleshy, skin tags of the nipple-areolar complexes. She underwent bilateral diagnostic excision of the lesions and macroscopically, both nipple specimens displaying polypoid lesions. Histological examination showed bilateral neurofibromas comprising skin with underlying dermal proliferation of bland spindle shaped cells with wavy nuclei. Immunohistochemistry confirmed the spindle cell proliferation to be neural in origin; positive for S100 and neurofilament and negative for cytokeratins. This was associated with florid smooth muscle proliferation. This case demonstrates a rare presentation of nipple-areolar neurofibroma occurring within the skin and nipple parenchyma. Our report considers the differential diagnoses of spindle cell proliferation within the dermis and subcutis of the breast and also other deeper breast spindle cell lesions that may involve the nipple. It aims to provide an approach to diagnosing these lesions examining the literature surrounding breast neurofibromas.

## 1. Introduction

Neurofibromas are rare, benign, peripheral nerve sheath tumours which can be a manifestation of neurofibromatosis type 1 (NFI). They typically develop on the trunk or limbs, affecting approximately 95% of individuals with NFI. NFI is caused by mutations of the NF1 gene leading to a nonfunctional neurofibromin protein, which usually acts as a tumour suppressor. Very few cases of breast neurofibromas are reported and were documented to predominantly occur on the nipple-areolar complex [1, 2].

## 2. Case Report

We present a case of a female aged 33 with a diagnosis of NFI. She presented with a longstanding history of bilateral nipple skin tags. No family history of NFI was documented. She had a history of hypothyroidism, epilepsy, and learning difficulties.

**Figure 1:**
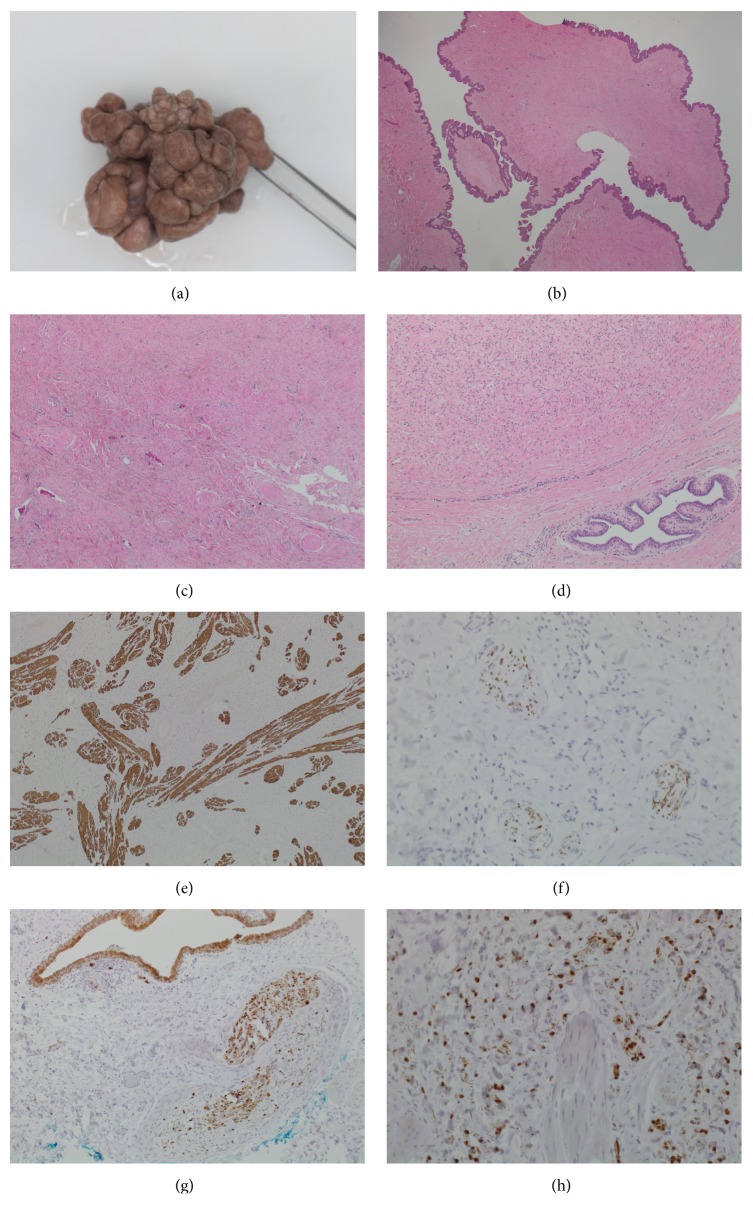
**Macroscopic and microscopic appearances of the nipple neurofibroma**. (a) Gross appearance of the largest lesion showing a pedunculated polypoid mass. (b) Overall H&E view showing a polypoid lesion covered by unremarkable squamous epithelium. (c) Low power H&E section showing bland spindle shaped cells interspersed with smooth muscle bundles of the nipple. (d) High power H&E section showing moderately cellular bland spindle cells extending close to a large mammary duct. There is no cytological atypia or mitoses. The adjacent mammary duct is benign and lined by inner luminal and outer myoepithelial cells. The lesional spindle cells show immunoreactivity for desmin (e), neurofilament (f), and S100 (g and h).

On clinical examination, bilateral, pedunculated, polypoid, and fleshy lesions were noted on both nipple-areolar complexes (Figure 1(a)). The right nipple lesion measured 60x55x35mm and left measured 25x25x25mm.

Microscopically, both specimens showed dermal proliferations of bland spindle-shaped cells with elongated wavy dark nuclei (Figures 1(b)–1(d)). No atypia, pleomorphism, necrosis, or mitotic activity was seen. Spindle cell proliferation extended close to a mammary duct (Figure 1(d)); however, it must be noted that these lesions did not arise from the breast parenchyma but included smooth muscle fibres of nipple and were in close proximity to mammary ducts. This can pose a diagnostic challenge when differentiating between spindle cell lesions arising from breast parenchyma which can also extend to the nipple.

Immunohistochemistry (IHC) supported the neural differentiation of the spindle cells with immunopositivity for S100 and neurofilament (Figures 1(f)–1(h)). Neurofilament usually stains axons in peripheral situations and not spindle cells. The proliferation was negative for a panel of cytokeratins including AE1/3 and p63.

Interestingly, large amounts of smooth muscle bundles were dispersed within the lesion. Desmin (Figure 1(e)) and smooth muscle myosin on IHC highlights the florid component of associated smooth muscle. This is thought to be a reactive process in response to the development of the neurofibroma.

## 3. Discussion

Spindle cell lesions of the breast are uncommon. The differential diagnosis is wide and histogenesis varied [1, 2]. Clinical history and IHC can provide invaluable information to aid histological diagnosis which can otherwise prove challenging. Breast spindle cell (BSC) lesions can be classified into benign BSC lesions and malignant BSC lesions [3, 6]. They can be entirely composed of spindle cells (pure) or mixed with other nonspindle cell components (mixed). In our case the bland BSCs were mixed with smooth muscle bundles with IHC positive for S100 and neurofilament (Figures 1(f)–1(h)) indicating the neural origin of the cells [7].

Four morphologically distinct variations of neurofibromas exist: plexiform, cutaneous, massive soft tissue neurofibromas, and localised intraneural tumours. On physical examination cutaneous neurofibromas, as presented in this case, have the appearance of flesh coloured papules or nodules of various size (Figure 1(a)). They can be pedunculated, dome shaped, or sessile and growth can be cosmetically disfiguring. As opposed to plexiform neurofibromas which are usually present at birth, cutaneous neurofibromas generally develop after adolescence [1–3, 6].

Neurofibromas are one of the two types of benign breast peripheral nerve sheath tumours (PNST), the other being breast schwannomas [1, 4, 8, 9]. Schwannomas are the most common of the PNST with a low malignant potential and can occur sporadically or in association with neurofibromatosis II (NFII) in 3% of cases [9]. Microscopically neurofibromas and schwannomas are typically composed of spindle shaped cells with elongated wavy nuclei with no features of atypia or areas of high mitotic activity. Neurofibromas do not display verocay bodies, Antoni A and B areas, nuclear palisading, or hyalinized thickening of vessels present in schwannomas. On IHC they display positivity for S100 protein. More diffuse and uniform S100 staining patterns are seen in schwannomas [6, 7, 9].

Differential diagnoses of benign breast spindle cell lesions include fibromatosis, myofibroblastic lesions, neurofibromas, schwannomas, nodular fasciitis, and scar tissue [4]. Malignant differentials include spindle cell metaplastic carcinoma (SCMC), malignant peripheral nerve sheath tumours (MPNST), malignant phyllodes tumour, and angiosarcoma and melanoma [6] (Table 1).

Myofibroblastoma are devoid of mammary epithelium and contain thick bundles of hyalinized collagen between the spindle cells. This was not observed in this current case. Myofibroblastoma is typically positive for desmin, SMA, CD10, and CD34, often positive for ER and androgen receptor, and negative for S100 [4]. Desmin positivity was also present in our case due to the smooth muscle component. Fibromatosis often displays collagen deposits among a fascicular pattern of spindle cells along with entrapped fat cells. Fibromatosis would show positivity for CD34, SMA, and B-catenin; however, it would be negative for S100. Nodular fasciitis, a rapidly growing lesion, arises from superficial fascia and comprises fibroblasts and myofibroblasts in a myxoid stroma with prominent vasculature. Positivity for SMA and vimentin and negativity for S100, desmin, and CD34 would be expected [4].

Neurofibromas can evolve into MPNSTs. It is therefore paramount that any evidence of malignancy such as necrosis, atypia, or mitoses is detected. MPNSTs are rare soft tissue sarcomas classified into three variants: glandular, epithelioid, and mesenchymal [10]. Distinguishing between neurofibromas and MPNSTs can be challenging as neurofibromas can occasionally exhibit some pleomorphic cells. Typically MPNSTs exhibit spindle cells in dense cellular areas with areas of high mitotic activity and loss of neurofibroma architecture. IHC typically shows reduced expression of S100 and CD34 with extensive p53 positivity [11].

SCMC is an important differential and can display a range of mild to severe atypia. Low grade fibromatosis-like spindle cell carcinoma is a variant of SCMC characterised by at least 95% of the tumour consisting of bland spindle cells [12, 13]. It can be difficult to differentiate from benign mimics. IHC of SCMC shows positivity for cytokeratins denoting their epithelial origin, not evident in our case. SCMC is often positive for basal cytokeratins and hence the importance of including basal markers such as p63, CK5, and CK14 in the panel if the diagnosis of metaplastic carcinoma is considered [5, 14]. Table 1 summarises the differential diagnoses of spindle cell lesions.

A useful panel of IHC for the diagnosis of spindle cell breast lesions includes broad spectrum and basal cytokeratins (to exclude metaplastic carcinoma), CD34 (negative in metaplastic carcinoma and positive in a variety of lesions), S100 (melanoma, neural differentiation, some carcinomas), HMB45 and Melan A (for melanoma), and desmin (for myoid differentiation). Other markers can be included depending on the morphological appearances (e.g., B-catenin in fibromatosis) [4, 7]. No single marker is fully specific and IHC should be interpreted in the light of the morphological findings.

## 4. Conclusion

Our case of bilateral nipple-areolar complex neurofibromas presenting in a female with NFI displays an unusual presentation and diagnosis within histopathology with a vast array of differential diagnoses. IHC plays a vital role in the diagnosis of breast spindle cell lesions. This highlights the importance of conducting a broad panel of immunohistochemical markers when breast spindle cells lesions are identified since there are significant differences in the management and prognosis of various differentials.

## Figures and Tables

**Table 1:** Differential diagnosis of common breast spindle cell lesions that may involve the nipple [3–5]. SMA: smooth muscle actin, ER: estrogen receptor, PR: progesterone receptor, DCIS: ductal carcinoma in situ, and CKs: cytokeratins.

**Differential diagnosis of common breast spindle cell lesions**		
**Benign**	**Histological Features**	**Immunohistochemistry**
Fibromatosis	Collagen deposition amongst spindle cells arranged in fascicles infiltrating fatty tissue. Lymphoid aggregates may be seen at the margins of the lesion.	Positive: SMA, B-catenin (80% nuclear), Negative: S100M CKs, p63, desmin,CD34

Myofibroblastoma	Irregular, nonencapsulated spindle cells with collagen bundles. Rare/No mitoses. No epithelial component seen.	Positive: desmin, SMA, CD34, ER, AR caldesmon, vimentin, Bcl2 and CD99. Negative: S100, CKs, p63, RB STAT6

Neurofibroma	Bundles of wavy spindle cells, elongated nuclei and narrow cytoplasmic processes.	Positive: Neurofilament, S100

Schwannoma	Similar to Neurofibroma but characteristic Verocay Bodies with nuclear palisading, Antoni A & B areas	Positive: Neurofilament, S100

Nodular Fasciitis	Fibroblasts with vesicular nuclei in myxoid stroma. Mitoses frequently detected. Rapidly growing lesion arising from superficial fascia	Positive: SMA, vimentin, Negative: S100,CD34,desmin, keratin

**Malignant**		

MPNST	Types: Glandular, Epitheliod, mesenchymal. Spindle cells in dense cellular areas of high mitotic activity. Areas of necrosis, nuclear pleomorphism	Positive: Neurofilament, S100 p53 Negative: EMA, keratin

Metaplastic Carcinoma	Malignant epithelial and mesenchymal areas. High mitotic figures, necrosis and nuclear pleomorphism. DCIS may be seen.	Positive: CKs, p63, vimentin, keratin Negative: CD34

Malignant Phyllodes	Leaf-like morphology, marked stromal cellularity and nuclear pleomorphism.	Positive/Negative: CD34 Negative: CKs

Melanoma	Can be composed of epitheliod +/spindle cells. Can have large pleomorphic cells.	Positive: S100, Melan A, Negative: CKs

Angiosarcoma	Haemorrhagic mass composed of epitheliod or spindle cells displaying vascular differentiation	Positive: CD31,CD34, keratin

## Abbreviations

NFI: Neurofibromatosis type 1
BSC: Breast spindle cell
PNST: Peripheral nerve sheath tumours
MPNST: Malignant peripheral nerve sheath tumour
SCMC: Spindle cell metaplastic carcinoma
SMA: Smooth muscle actin
IHC: Immunohistochemistry.
